# The reconfiguration of biobanks in Europe under the BBMRI-ERIC framework: towards global sharing nodes?

**DOI:** 10.1186/s40504-020-00105-3

**Published:** 2020-10-01

**Authors:** Violeta Argudo-Portal, Miquel Domènech

**Affiliations:** Barcelona Science and Technology Studies Group (STS-b). Department of Social Psychology, Universitat Autònoma de Barcelona, Campus de la UAB. Bellaterra (Cerdanyola del Vallès), 08193 Barcelona, Spain

**Keywords:** Biobanks, Europe, Infrastructure, Global, Biomedical research, Mediator, Spain, Network, Scalability, Science and technology studies (STS)

## Abstract

Freezers with biospecimen deposits became biobanks and later were networked at the pan-European level in 2013 under the Biobanking and BioMolecular Resources Research Infrastructure—European Research Infrastructure Consortium (BBMRI-ERIC). Drawing on document analysis about the BBMRI-ERIC and multi-sited fieldwork with biobankers in Spain from a science and technology studies approach, we explore what biobanks are expected to do and become under the BBMRI-ERIC framework, and how infrastructural transitions promote particular transformations in biobanking practices. The primary purpose of biobanks in Europe is presented as being to become mediators in contemporary biomedical research (global sharing nodes) distribution, and distributed nodes of samples and their associated data. We argue that infrastructural transitions are complicated and heterogeneous, giving rise to unattended local concerns on adjusting their practices to fit into the BBMRI-ERIC framework, even for non-members, as the case of Spain illustrates, where “old practices” of collection and storage are questioned. In this article, we aim to encourage qualitative studies to explore the lags between pan-European policies and prospects, different contextual interpretations, and biobanking reconfigurations as an opportunity to explore what that lag is made of (e.g. tensions with “old practices,” unresolved conflicts with the national agendas, reservations on a possible centralization of the biobanking practices by regional biobanks, lack of funding, etc.). Such research could enrich not only policy guidance, but also the understanding of technoscientific infrastructures’ scalability.

## Introduction

In the late 1990s, several countries started to establish “nationally delimited, population-based genetic databases, more commonly known as population biobanks” (Mitchell and Waldby 2010, 332). These biobanks were framed under the narrative of national resources (Cambon-Thomsen et al. 2003) and population national branding (Tupasela 2017b). Biobanks became national projects involving articulations between the state, the public, the scientific community, and economic agents. The spread and rise of national biobanks run chronologically and in parallel with the last phase of the Human Genome Project (HGP), a large-scale biology project that has been considered a transformative agent of biology and medicine throughout a “big science” approach (Collins et al. 2003; Hood and Rowen 2013). In April 2003, at the completion of the HGP and the start of the referred to as the post-genomic age (Gottweis and Lauss 2010), a benchmark for “thinking bigger,” which is inseparable from bioresearch and thus from biobank projects, had germinated. “Thinking bigger” can be understood as the result of a shift in biological research to examining the larger picture in order to research “what life is: the mechanisms, pathways, and systems” (Hadley 2004, 236), and discerning complex connections that, on a smaller scale, could not be detectable. A global competition within pharmacogenomics has also encouraged this way of “thinking” (Pálsson 2007), and has furthered a commercial imperative for these projects. A part of “thinking bigger” in biomedical research draws upon human biospecimen repositories, such as biobanks, whose procedures called for harmonization to allow the general use of samples, their associated data, and the services required to ease the post-HGP biomedical research.

On this matter, it is important to note that biobanks as national projects were formally established right on the edge of the 20th to the twenty-first century, in a time when the previous frame of the “taken-for-granted” (Wynne 2007) trust in Science, with a capital S (Latour 1999), was questioned. Indeed, expectations linked to biobanks gave rise to ambiguities and uncertainties (Stephens and Dimond 2015; Tutton 2010), conceptualizing them as ethical problems (Hoeyer 2008), and, as such, biobanks were framed under the ELSI (ethical, legal, and social issues) agenda. As other scholars have noted (Balmer et al. 2015), the ELSI framework tends to bifurcate realities, “splitting-up systems of reality” (Whitehead 2006) such as science and society. In biobanking, ELSI approaches mainly revolve ethics challenges regarding donors (Kasperbauer et al. 2018; Ursin and Stuifbergen 2018), informed consent (Boniolo et al. 2012; Kaye et al. 2015), legislative frameworks for sample and data use (Zika et al., 2008), donors’ attitudes and participation (Lipworth et al. 2011; Bossert et al. 2017), etc. But, as Cadigan et al. (2013) noticed, pay little attention to biobankers practices and perspectives.

Terminology varies when referring to biomedical depositories where “bio” samples are stored: repositories, biorepositories, collections, banks, cryobanks, biobanks, etc. According to Tsing (2015, 29), “to use category names should be a commitment to tracing the assemblages in which these categories gain a momentary hold”. So, it was during the second half of the 1990s when the term “biobank” started to be used as the preeminent reference to human-based repositories that collected and stored human biospecimens and their associated data. Biobanks can be classified in different ways based on tissue type, purpose, ownership, group of participants, or size (Arampatzis et al. 2016). While there is a general shared picture of what a human-based biobank is, definitions in academic papers, guidelines, and policy reports are wide, varied, and nuanced (Milanovic et al. 2007; Hewitt and Watson 2013; Shaw et al. 2014). Mayrhofer (2011) asserted that the term “biobank” is an umbrella term, but others have suggested that the concept of a “biobank” might be too singular (Hoeyer et al. 2017). The approaches, in all their diversity, seem to recognize the complexity and hybridity of the forms involved in biobanking (Romero-Bachiller and Santoro 2018; Tupasela et al. 2017). Tsing (2015, 293) explained, “[I] need names to give substance to noticing, but I need them as names-in-motion.”

However, harmonization processes and procedures allowed the transformation of researchers’ fridges or hospital anatomical pathology deposits into the technoscientific infrastructures we call biobanks, which later would be proposed to become virtually networked by the European Commission in 2013 as the Biobanking and BioMolecular Resources Research Infrastructure—European Research Infrastructure Consortium (BBMRI-ERIC). This pan-European initiative placed biobanks “in a process of transition from individual research tools to complex international research infrastructures. This process is not an ordered and homogenous change, but rather a complex and problematic transition” (Meijer et al. 2012, 497). Scholars studying infrastructures have noted (Harvey et al. 2016) that a linear development cannot be assumed. Infrastructural politics generate complications as are shaped by multiple and distributed interactions, and so do infrastructural transitions. There are some complications that highlight the relevance of Tupasela’s (2017a, 190) observation: “There is a need to develop a more nuanced theory of biobanking politics, where the interests of scientists who control samples and data are better understood and recognized in relation to the more normative political expectations associated with sample-and data-sharing set out in polices.” In this paper, following a science and technology studies (STS) approach, we point to how these infrastructural transitions and their complications take part in biobanks’ social worlds, conceptualizations, and practices. We aim to promote further inquiry into how situated reconfigurations in biobanking might note a lag with the policies and prospects at the pan-European level, and explore what the lag is made of in different contexts (e.g. tensions with “old practices,” unresolved conflicts with the national agenda on biobanking, reservations on a possible centralization of the biobanking practices by regional biobanks, etc.).

Assuming that “[t] he question ‘what is infrastructure’ must therefore be addressed, and experimented with, in registers at once conceptual and empirical” (Harvey et al. 2016, 6), our inquiry combines both, but it stems from our fieldwork about biobanking in Spain, which is focused on the practices and concerns of staff in different Spanish biobanks. We identified a strong presence of the BBMRI-ERIC, despite Spain’s not being a member of such infrastructure (as of June 2020). Therefore, we decided to develop a document analysis to explore how biobanks are conceptualized by exploring what they are meant to do at the pan-European level as presented in science policy prospects and reports. Next, following Spanish biobankers’ aspirations to be part of the BBMRI-ERIC, we show how reconceptualizing biobanks to fit under the pan-European framework is translated into tension with “old practices,” in particular regarding the collection, storage, and stockpiling of samples. We argue that greater attention and qualitative research focusing on biobankers practices and country-specific concerns are needed in combination with scrutinizing policy documents to identify and to describe unattended interpretations, shifts, and parallelisms between pan-European policy prospects on infrastructures and their doings at different levels.

## Materials and approach

The argument of this paper is based on two sources of data: qualitative fieldwork and documents. We draw on 16 months of multi-sited fieldwork, between 2018 and 2019, with biobank staff in Spain,^1^ including participant observation in workshops, conferences, newspaper follow-ups, and 14 semi-structured interviews with biobankers from seven different biobanks that lasted between 45 min and 2 h. We interviewed biobanks’ directors, lab technicians, administrative staff, researchers, security guards, to explore their perspectives and practices. We tape-recorded and fully transcribed 12 interviews. The other two interviews were not fully-transcribed. Two interviewees did not allow us to tape-record the interview (both of them biobank directors). One of these two did not allow us to quote the content of his interview, following the informed consent opt-out options. In these two cases, we took notes. We omitted the biobanks’ names and specific locations, and assigned pseudonyms to the interviewed personnel, keeping only their professional status to situate their comments in the context of their positions. In our qualitative fieldwork and documentation of the Spanish context, the BBMRI-ERIC appears as a milestone to reach, an institutional aspiration, and a model to conceptualize and to articulate biobank practices. Therefore, considering our findings, we decided to approach the BBMRI-ERIC through document analysis to better understand the current state of affairs at the pan-European level, to see how biobanks’ primary purpose is formulated and presented in such documents under the infrastructure that Spanish biobanks take as a reference.

We reviewed documents dating from 2002, when the European Strategy Forum on Research Infrastructures began, to the current day (early 2020). For the first period (2002–2013), analysis was primarily based on two reports, the “Biobanks in Europe: Prospectives for Hamornisation and Networking” (2010) by the JRC of the European Commission and “Biobanks for Europe: A challenge for governance” (2012) by the Directorate-General for Research and Innovation. We also consulted Community Research and Development Information Service (CORDIS)^2^ reports and publications regarding the Seventh Framework Programme for Research and Technological Development (FP7) between 2007 and 2013, and Reports on the European Strategy Forum on Research Infrastructures (ESFRI). For the second period (2013–2019), we incorporated BBMRI-ERIC documents, such as annual reports, statutes, deliverable reports, press releases, Biobanks Europe Magazine, and newsletters. For the matter of this paper, the focus is placed in the second period, 2013–2019. In the following section we explore how biobanks are presented under the BBMRI-ERIC. Considering the way the BBRMRI-ERIC assemblage is presented (and thus biobanks) has crucial implications because, as Puig de la Bellacasa (2011, 87) put it, “how we present things matters.”

## Scaling up biobanks: the BBMRI-ERIC

The European Strategy Forum on Research Infrastructures (ESFRI) was set up in 2002 following an EU Council call to support and encourage multilateral projects in the EU and internationally, aiming to develop and improve different research infrastructures. Since then, the ESFRI roadmap has been brought up to date in 2008, 2010, 2016, and 2018, and it is expected to be updated in 2021. In this context, the BBMRI-ERIC, as one of the projects within the pan-European research infrastructures’ strategy in the 2006 roadmap began its preparatory phase in 2009 and ran until 2011. During this phase, in 2010, the JCR Scientific and Technical Report published “Biobanks in Europe: Prospects for Harmonisation and Networking.” This report served as a call for attention for what was yet to come in biobanking, as well as a formal recognition of the overwhelming harmonization challenges to be faced not only at the pan-European level but also at the national and regional levels, as other researchers have addressed (Chadwick and Strange 2015). The BBMRI-ERIC acquired its legal status in 2013 and, as of June 2020, includes sixteen full member states and four observers. The main goal of the BBMRI-ERIC is described in its Statutes as follows:

BBMRI-ERIC shall establish, operate and develop a pan-European *distributed* research infrastructure of Biobanks and Biomolecular Resources in order to facilitate the access to resources as well as facilities and to support *high quality* biomolecular and medical research. (BBMRI-ERIC 2016, emphasis added)

Scholarly publications have followed the establishment of the BBMRI-ERIC’s announcing its potentialities as “a resource for pharmaceutical and life sciences industries” (Van Ommen et al. 2015), a gateway to access collections for the European research community (Mayrhofer et al. 2016), and the “[l] aunch of an infrastructure for Health Research” (Litton 2018). Other scholars have focused on analyzing the challenges faced during the preliminary phase (2009–2011). For instance, Tamminen (2015) identified two key challenges in that period: first, mapping existing biobanks and second, concerns related to language harmonization, which led to the creation of a “lexicon for global biomedical research.” The establishment of the BBMRI-ERIC and networking biobanks at the pan-European level requires changing some of the ways that biobanks used to work, involving a complicated transition (Meijer et al. 2012) and exacerbation of already existing governance challenges.

Overall, the BBMRI-ERIC intends to integrate already existing research infrastructures and research communities from the field of life sciences in Europe, taking advantage of information systems and boosting biobanks’ capabilities for biomedical and drug research. Bioinformatics’ combination of molecular biology investigation and computer science has promoted an emergent way of understanding diseases that depends upon infrastructures that build and maintain large-scale networks of databases, establishing and integrating research infrastructures for the “virtualization of biological work and biological objects” (Stevens 2013). From the scientific community perspective, thicker networked infrastructures are crucial in order to use the biomedical capabilities that biobanks can provide for what is often referred to as “precision medicine” as well as for research on biomarkers and their resulting potential treatments.

The BBMRI-ERIC does not consist of the establishment of a new physical European biobank, but the creation of an active network among existing biobanks and bioresources. Network membership requires an economic contribution by the member state, and then, each member state designates a national node that will coordinate the existing biobanks in that country to scale-up their bioresources to the pan-European level. This network brings forth tools such as the BBMRI-ERIC directory, which enables searches of the bioresources of European biobanks by applying several search filters and variables. Expansion occurs through scale-up, as in many other capitalist processes (Tsing 2015), scalability is at stake. Still, the success of scalability or the possible leaks and failures call for situated research at the national and sub-national levels to account for “the links between the scalable and nonscalable” (Tsing 2012, 154).

Scaling up digitally as opposed to the creation of a bigger physical enterprise can be addressed in its specifics through the scaling dynamics identified by Sassen (2007) regarding global digital formations and Tsing’s (2012) nonscalability theory. The BBMRI-ERIC infrastructural assemblage gathers features that characterize what Sassen (2007) calls the “networked sector,” while simultaneously undergoing deterritorialization and territorialization processes that feed on each other. Harmonized procedures and digital directories may work as critical hinges here, allowing integration, contraction, and expansion as much as deterritorialization and territorialization. Tsing’s nonscalability theory proposes that “instead of taking scalability for granted as a necessary tool of progress, nonscalability theory attends to the work of contingency and failure. Nonscalability theory shows us scalability in action” (Tsing 2012, 148). Therefore, scaling up cannot be approached as an inevitable occurrence, but an ongoing work that generates numerous frictions. We will exemplify one of such frictions further on regarding biobanking practices in Spain, but before this, let us explore biobanks under the BBMRI-ERIC framing.

## Biobanks as mediators

The BBMRI-ERIC case provides a set of practices, proposals, and expectations to help readers grasp what biobanks are meant to do in Europe. This particular infrastructure reveals an effort to digitally harmonize and integrate different biobanks. According to Article 1 of the BBMRI-ERIC’s Statutes (2016) biobanks, in plural, are “collections, repositories and distribution centers of all types of human biological samples … and/or related data … contribute to the understanding of the physiology and diseases of humans.” Under this infrastructure, integration aims to facilitate sharing bioresources, such as biospecimens and associated data, that are expected to contribute to biomedical research and clinical care, rather than being banked *in aetérnum*. Taking care of a biospecimen implies making it available in its best quality (i.e., the sample and its associated data) and for this, the BBRMRI-ERIC provides the infrastructure to scale-up biobanks by creating a federated network.

Hoeyer et al. (2017, 388) observed that “the concept of ‘biobank’ might be too singular (one set of samples in one place), too static to capture the sense of flow (it indicates accumulation), and too informed by one type of purpose (research) to capture all the involved flows and uses”. Paying attention to flows requires to be aware that flows preceded the assemblage, albeit without the formal infrastructure required in order to be used efficiently, which is precisely the aim of the BBMRI- ERIC. Star and Ruhleder (1996) explained that infrastructures aim to resolve the tension between the local and the global, and, to do so, connections are made, for instance, through bioresource directories. When scaling-up digitally, the connections involved in this practice make infrastructures pop-up, or become visible. Connections are also stressed in the BBMRI-ERIC Report (BBMRI-ERIC 2013, 22) where it is said that biobanks are expected to act as an **“**intermediary” between donors/participants, scientists, patients, hospitals, and so on. If recognition of biobanks’ mediator position is central to reaching their purpose, increasing their visibility becomes a need in order to make the assemblage work. For this reason, it was not surprising to read while writing this paper that the title of the current Action Plan of the BBMRI-ERIC 2019–2021 is “increasing the visibility of biobanks and sample collections” (BBMRI-ERIC 2019).

However, *intermediary* is not an innocent term. STS scholars are wary of the differences and nuances between intermediaries and *mediators* (Serres and Latour 1995; Latour 2005). Is biobanks’ only purpose to allow access to samples and data or do they also redefine what they collect and share? An intermediary, according to Latour (2005, 39), “transports meaning or force without transformation”, working as a black box or a unit; while mediators are “endowed with the capacity to translate what they transport, to redefine it, redeploy it, and also to betray it” (Latour 1993, 81). Mediators can betray, but also care. In fact, biobanks tend to be endorsed as caretakers of the samples and their associated data. Biobanks’ curating practices and knowledge production on biospecimens shape and redefine how science is made.

As mediators and not mere intermediaries, biobanks in the BBMRI-ERIC network express the aforementioned “thinking bigger.” In this case, “thinking bigger” does not entail “making it bigger” as in physics (Vermeulen 2016), like in the case of the Large Hadron Collider, but doing it bigger by digitally scaling up infrastructures, collaborations, and networked services. Connections are what enable this “thinking bigger” and make it global (Serres 1994). Biobanks act as mediators when their central purpose is to distribute samples and their associated data, instead of just accumulating them. Regarding biobanks as mediators, we have identified three different practices that characterize biobanks under the BBMRI-ERIC logic: globalizing, sharing, and localizing.

### Globalizing

Globalizing is made of connectivity and virtualization. The pan-European network of biobanks builds on the connection of several distributed infrastructures, bearing upon directories, datasets, freezers, samples, computers, workers, lab devices, donors/participants, etc. Indeed, it is this infrastructural assemblage that enables multiscalar practices and global effects. We can describe, thus, how fridges in offices and hospitals were compiled and relocated to a common space with other freezers, which were connected to computers and registries of digitized data. Informatics capabilities, standards, and harmonization processes enabled a network of freezers with biospecimens and their associated data, but also a network of infrastructural labor. Connections are what make the network *global* (Serres 1994).

Additionally, biobanks in Europe through the BBMRI-ERIC brought about a process of virtualization. According to Lévy (1998, 29), virtualization is based on a breakdown of spatiotemporal coordinates, “incorporating temporal unity without spatial unity”. This breakdown does not make spatiotemporal coordinates fully independent because they still bond to the physical; virtualization does not entail disappearing or dematerialization (Lévy 1998). Hence, we approach *global* in the sense of generative connections rather than as a universal or territory-based conceptualization. Collier and Ong (2005, 11) have posited that global phenomena “have a distinctive capacity for decontextualization and recontextualization, abstractability and movement, across diverse social and cultural situations and spheres of life”, and so do these networks.

### Sharing

The goal of collecting samples and their associated data under high-quality standards aims to engender and distribute the best quality biospecimens and associated data (bioresources) for biomedical research projects. This emphasis on “quality” here is mainly a focus on enabling replicability, and for that end, work is needed to ensure that, as Tarkkala (2019, 36) has also observed, “the samples stored should be what they are said to be”—something that has not been that mundane or common. *Sharing,* and not banking as accumulation, involves paying greater attention to what is presented as biobanks’ current main function: visible–physical recipients of biospecimens. And, yet, also abstract mediated data-transmitters that enact bioresources’ mobilities. In biobanking, biospecimens are samples, but they are also meta-data: “biological exchanges [that] informationalize without dematerializing” (Thacker 2005, 11). Milanovic et al. (2007) analyzed the plurality of *exchange* regimes present in biobanking, which we acknowledge, but for this paper, we have decided to use the word *sharing* instead. While the word “exchange” tends to refer to a more evident bidirectional relationship, in biobanking, the relationships consist more of gift-giving, which engages with highly mediated modes of reciprocity. The BBMRI-ERIC report reads:

Typically, biobanks are viewed as a “public good”: a shared resource to which individuals contribute through their blood donations and that will, eventually, result in a reciprocal benefit in the form of better and more effective medical treatment options. (BBMRI-ERIC Report 2013, 38)

We propose to work with the word “sharing” as it better condenses the idea of the action that takes place in biobank relationships, one with a less clear trajectory. In addition, sharing often involves dividing things into portions,^3^ which definition better relates to biobanking. This definition of “sharing” also makes explicit the logic of the phrase, “sharing is caring,” as sharing is a way to take care of the sample—by making it available for use. However, this logic also raises concerns. Therefore, we will not talk about a vision of innocent and romantic share-care, as Puig de la Bellacasa (2011, 100) notes: “a way of caring over here could kill over there.” Nonetheless, sharing is critical. So we must turn to ask: Who shares what, for whom, and for what? Following the features of digitally scaling-up biobanks, instead of creating a bigger biobank, commensurability is put aside in this reflection. Biospecimens and their associated data become singularities of a “troubling multiplicity of instances” (Strathern 2004).

### Localizing

Globalizing and sharing evoke images of centrifugal forces, yet biobanks’ materiality matters. Therefore, we also recall centripetal forces, approaching them as localizing practices. Contrasting with the virtualization of samples and sharing practices, we take as a reference Kaye’s (2011) conceptualization of biobanks as *nodes*, from which data flows internationally through local researchers and institutions. *Node* implies an anatomic differentiated material mass—for instance, biobanks’ deposits, lab gloves, computer servers, and the material form of biospecimens are the aspects that can be readily differentiated from the whole research infrastructure or the imbrication. The anatomical allusion behind the notion of *node* provides physicality and avoids approaching digitization as lacking materiality. Also, biobanks in the BBMRI-ERIC infrastructure and framework compare favorably with other uses of the global node concept, such as Harvey’s (2007) use of the concept *nodes* in a global network in the case of the London Gold Fix, as an active and essential local connecting point. Nodes localize materials and ways in which others care, as much as they generate “matters of concern” (Latour 2004) regarding national jurisdictions, data privacy, replication controversies, etc. Without lab technicians processing the samples, server stations, office workers at the donor call centers, or security personnel attending around the clock to beepers connected to the frozen deposits, there is no biobank nor, hence, a network of biobanks. However, it is necessary to note that the BBMRI-ERIC defined the entities that coordinate biobanks in different countries as nodes and not biobanks themselves. Therefore, this analysis does not follow the BBMRI-ERIC use of the term but plays with it.

Following our analysis of the BBMRI-ERIC, we have engaged in an exercise of creating a compound noun as a descriptive device that will contribute to a better understanding of biobanks in the current European context. We take Kaye’s (2011) proposal of defining biobanks as nodes, but we go a step further, defining a biobank as a node of a global network—a *global sharing node*. This reflective proposal avoids the commensurability attached to other adjectival word choices such as “large-scale” or “big.” In this compound noun, size is set aside but not disregarded and, instead, we emphasize multiscale mobilities and localities, scalability and nonscalability. The term *global sharing nodes* accommodates current biobank practices and enables readers to grasp what biobanks are presented to do and become after the establishment of the BBMRI-ERIC infrastructure in Europe. Situating biobanks in the network as *global sharing nodes* is an exercise that purports to indicate their reconfigurations, after the establishment of the BBMRI-ERIC in 2013.

## The nodes to be: parallelisms, reconfigurations, and aspirations from Spain

In Europe, biobanks’ governance, organizational features, and funding actors vary in every country (Meijer et al. 2012). In the case of Spain, biobanks are under the umbrella of the Spanish National Healthcare System’s strategic agendas for the promotion and improvement of universal and public healthcare. Indeed, the Royal Decree 1716/2011 that specifically legislates biobanks highlights their “public service vocation,” whose base is to provide quality samples to the scientific community. The Spanish Biobank Network (RNBB) was created in 2009, and between 2011 and 2013 the majority of biobanks were formally established and registered, following the aforementioned decree. Indeed, 88 out of 99 biobanks were registered in the National Registry of Biobanks (as of July 8, 2020) before 2014. Currently, the Spanish Biobank Network includes hospital-based biobanks, regional networks, research institutes, and university biobanks from all over the country. The efforts of the national network in its first decade have been placed on working in a coordinated but decentralized manner, and in the creation of a sample catalog and a unique window for sample requests.

Despite Spain’s not being a member of the BBMRI-ERIC, the timeline of biobanks’ establishment runs in parallel with that of the BBMRI-ERIC at the pan-European level. These parallelisms and presence can be tracked prior to 2013 when the BBMRI-ERIC acquired its legal status. In 2010, one of the principal representatives of the biobanking community in Spain participated in the BBMRI Stakeholder’s forum (2010), and in July 2020, if you check the Spanish Biobank Network Wikipedia entry “vision and mission” (whomever has edited it) states that it has a similar design to the preliminary BBMRI project. We have already pointed to how the BBMRI-ERIC Report (2013) situated biobanks as “intermediary agents,” a position that is currently mirrored in Spain. In February 2020, the Spanish *New Medical Economics Journal* published “Biobanks: Connectors between patients, researchers, and physicians.” Therefore, we argue that the BBMRI-ERIC has been serving as a model for conceptualizing biobanks’ work and reconfiguring their practices since their formal establishment in Spain. But, how is the BBMRI-ERIC interpreted, and why is it important to be part of it from the biobankers perspective? Which implications entail making biobanks become *global sharing nodes* for biobankers practices?

During fieldwork, biobankers made explicit that being part of the BBMRI-ERIC is something they have requested for a long time, but that it has been postponed due to the membership fees required to become a member. After the 2018 IX Spanish National Conference of Biobanks, media reported that for the biobanking community in Spain, the way to fit into the BBMRI-ERIC was “the principal challenge of the future,” as pointed by the president of the biobanks’ scientific committee during the 2018 conference. The headlines of some newspapers’ editorials in the autumn of 2018 wrote: “Spanish biobanks search how to fit into a European structure” (Diario Médico, November 18, 2018) or “Spanish biobanks are looking forward to being part of the primary European network” (La Vanguardia, November 18, 2018). Considering that the BBMRI-ERIC presents biobanks as global sharing nodes, this notion of “how to fit” in Spain has turned into working on the coordination of the national network, but also it has highlighted concerns regarding the underuse of samples due to “old practices” on collections, storage, and stockpiling. Therefore, the BBMRI-ERIC serves as a classical “Europe-is-the-solution” kind of argument (Gasset in Narotzky 2016) for Spanish underfunded technoscientific infrastructures, but also it provides a platform for collaborations, and therefore for the use of stored samples.

In general, one can easily see how many samples have been transferred for research by a particular biobank or a total calculation gathered by the national network (as of July 8, 2020, the Spanish Biobank Network website announces that 629,592 samples have been transferred by the network). Yet, the rotation rate, the number of samples collected and transferred per year are not that transparently publicized nor easy to access. Indeed, we were unable to collect such data in most of the cases. We understand the reasons that might be behind these strategic decisions. Indeed, the underuse of biospecimens has also been pointed to as a neglected issue in biobanking in other contexts (Cadigan et al. 2013; Stephens and Dimond 2015). However, during fieldwork, we found that biobankers did not neglect this issue. In an interview with a biobank researcher and administrative staff from one of the biggest biobanks in Spain, they explained that stockpiling samples of rare diseases is justified due to scarcity logic, but not necessarily in other cases. As an example of the current discussions among biobankers in Spain, we present the following excerpt:

- “A register (of potential donors) makes the biobank more sustainable” says Pedro, a biobank researcher. “This way, you can identify potential donors of samples for biomedical research. Then, when there is a project request, you know the specifics of the sample and the format you need, instead of storing samples without knowing what purpose they will be used for. This way, you avoid having thousands of samples in the freezer. Storing samples means that some can be damaged, can fall …”
- “Or that they (samples) will never be used,” intervenes Juana, administrative staff from the biobank.
- “Right, that is also what I mean when I refer to the biobank’s sustainability,” points out Pedro.
- “But do biobanks store a lot of samples? I thought the main purpose was to make them available for research, to be used in biomedical research projects,” the interviewer asks.
- “I remember a rotation index from a few years ago … and the majority of the samples were not getting out, and you cannot store them forever and ever. At some point you need to stop … [i] t is impossible to maintain a biobank if 90% of the samples become stock. It should be the other way around—10% stock and 90% transfers,” answers Juana. (Interview, October 2018)

What the interviewees discussed is how the storage and stockpiling of samples, which has been portrayed as the primary function of biobanks, do not necessarily facilitate biobanks’ main purpose today as distributors of samples, as *global sharing nodes*. This tension cannot simply be answered under scarcity or a selfish logic. Instead, what the interviewees are explaining is that if biobanks aim to become distributors of samples rather than warehouses or underused libraries, then, sample collection needs to be reconfigured. Switching to a prospective collection of samples (when possible) for specific projects would reduce stock, as Pedro explained. Thus, the how, what, and when to store become basic and relevant questions to be formulated in order to promote anticipation or preparedness, rather than banking and waiting for a hypothetic future use. This shift requires a reconfiguration of the “old practices” (hoarding samples) that have characterized biobanking, at least in Spain.

The excerpt summarizes what biobanks are no longer for Spanish biobankers, and shows the emergence of infrastructural complications: on one side, the practice of storing/banking is under scrutiny, and thus biobanks’ main function is also; on the other side, a registry of potential donors of biobank samples is presented as a possible mechanism to reduce the underuse of samples. Data accumulation is then presented as a tool for biobanks’ sustainability. We observe that while data follows a logic of accumulation (Hoeyer 2018), in this case, it takes the form of a registry of potential donors, biospecimens do not necessarily follow this rationale. Sorting out how to become global sharing nodes mirroring the BBMRI-ERIC in the Spanish context is entangled with reconfiguring banking practices to address the underuse of samples, among other things. However, reconfiguring biobanks’ practices might vary cross-nationally from non-members of the BBMRI-ERIC. For example, a visit to the coordinator center of the Danish National Biobank or/and a consult of their website shows that sample accumulation and storage is at the forefront. Therefore, not all countries in Europe might be interpreting becoming global sharing nodes in the same way. And, not all biobanking communities might even be committed to fitting under the BBMRI-ERIC prospects.

## Conclusions

Freezers and deposits with samples became biobanks and later were called to be networked at a pan-European level to expand their potential as distributors of samples and their associated data. Sample and data collection, biospecimen science, the curation of the sample, and—most importantly—making these bioresources available for biomedical research are some of the practices involved in biobanking. Biobanks’ promises and expectations as caretakers of human biospecimens and their associated data are related to a particular understanding of diseases and treatment development, such as how medicine and healthcare are framed in a specific moment and context. Biobanks’ virtualization or data-sharing emphasis is part of a broader framework in which “thinking bigger” in the life sciences and precision medicine are conceived mainly as data-driven medicine (Prainsack 2017). Thus, contemporary biobanks under the European framework are mediators for biomedical research, engendering and articulating particular modes of reciprocity, knowledge production, and forms of “biovalue” (Waldby 2000).

Infrastructural transitions have implications on how biobanks’ purpose is presented, and therefore, is transformed or how their practices are put in question. In Europe, these transformations are led by the BBMRI-ERIC, which policy prospect connects biobanks at a pan-European level, as a “networked sector,” re-articulating biobanks challenges and practices. We identified that the establishment of this infrastructure highlights biobanks as mediators between researchers, patients, hospitals, physicians, and so forth. Three practices summarize how the BBMRI-ERIC scales-up biobanks as mediators: globalizing, sharing, and localizing. We proposed that this is best expressed through the compound noun *global sharing nodes*: *Global*, as made by connectivity and virtualization; *sharing*, to look at the main expected action; and *nodes*, to stay with biobanks’ materiality and local practices. Tracking what biobanks are expected to be and do, also indicates what are not supposed to do and be any longer. Biobanks’ transformations under the BBMRI-ERIC framework have implications even for non-members, as we have shown in the Spanish case. Does the establishment of the BBMRI-ERIC have a presence in other non-members states? If so, with what implications or interpretations? And among current members, what do members of the BBMRI-ERIC do in practice?

In Spain, where the biobanking community aspires to be part of the BBRMI-ERIC, biobank practices are called to be transformed to better fit in what biobanks are expected to do nowadays. We incorporated this particular case to show a lag between biobanks’ policy framework prospects at the pan-European level, and interpretations and infrastructural complications regarding biobanks’ old practices, that is, what scalability entails. A grounded guidance at the policy level on how to transition towards becoming *global sharing nodes* is needed in order to address local concerns on the emergence of biobanks’ infrastructural and economic fragilities under this passage, as the example of the personnel concern on biospecimen underuse and the reconsideration of storing practices illustrate. In this regard, empirical qualitative research on the lags between pan-European policy prospects and biobanks’ transformations in different contexts are highlighted as valuable in understanding not only these infrastructural transitions but also scalability in contemporary technoscientific projects.

## Data Availability

The majority of the documents analyzed in this paper are available at: https://www.bbmri-eric.eu/publications; https://cordis.europa.eu/en; https://www.esfri.eu/ and https://op.europa.eu/en/publication-detail/-/publication/629eae10-53fc-4a52-adc2-210d4fcad8f2.

## Abbreviations

BBMRI-ERIC: Biobanking and Biomolecular Resources Research Infrastructures European Research Consortium
CORDIS: Community Research and Development Information Service
ELSI: Ethical, Legal, and Social Issues
ESFRI: European Strategy Forum on Research Infrastructures
HGP: Human Genome Project
RNBB: Spanish Biobank Network
STS: Science and Technology Studies
FP7: Seventh Framework Programme for Research and Technological Development

## Footnotes

1 Quotes from interviews and documents in Spanish have been translated by the authors.

2 CORDIS is the European Commission’s primary public repository and portal to disseminate information on all EU-funded research projects and their results.

3 The main entry of the verb *share* in the Oxford Dictionary reads: “have a portion of (something) with another or others.” Two sub-entries refer to it but with other verbs such as give, use, occupy, and enjoy (something). Oxford Dictionary Online: https://www.lexico.com/en. Accessed 28 August 2019.
